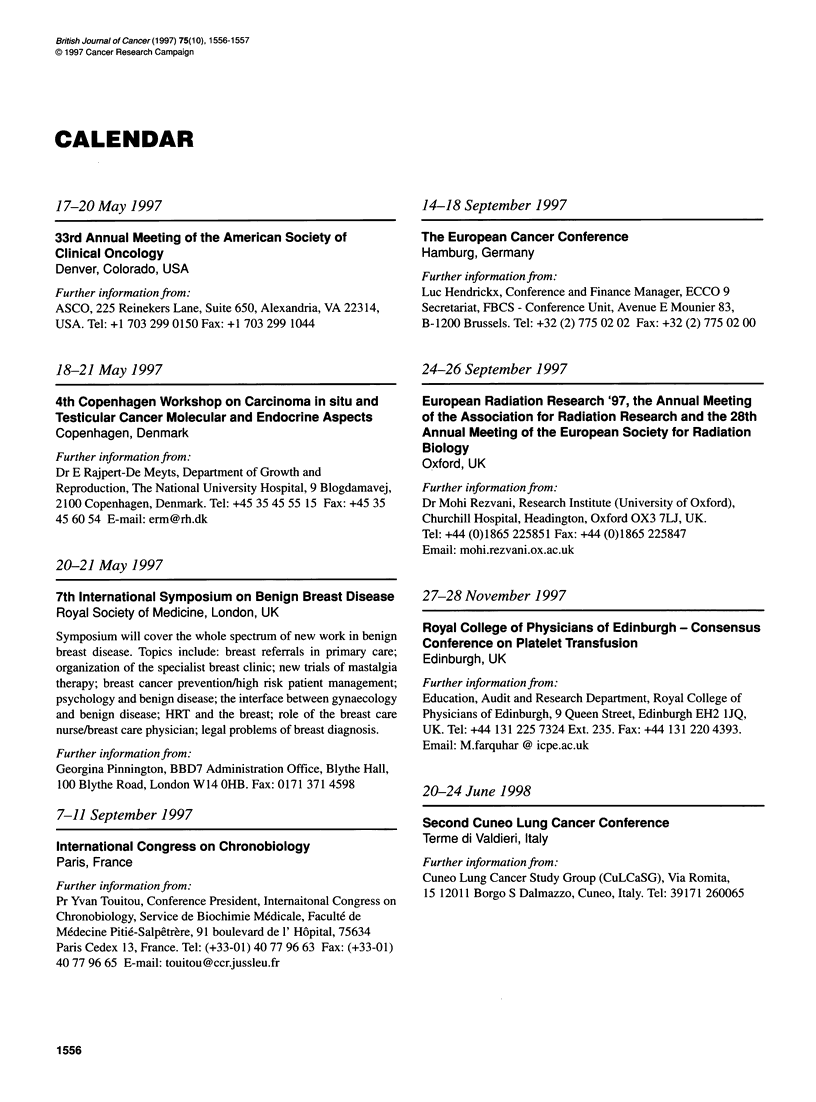# Calendar

**Published:** 1997

**Authors:** 


British Journal of Cancer (1997) 75(10), 1556-1557
© 1997 Cancer Research Campaign

### CALENDAR

*17–20 May 1997*

**33rd Annual Meeting of the American Society of Clinical Oncology**
Denver, Colorado, USA

*Further information from:*
ASCO, 225 Reinekers Lane, Suite 650, Alexandria, VA 22314, USA. Tel: +1 703 299 0150 Fax: +1 703 299 1044

*18–21 May 1997*

**4th Copenhagen Workshop on Carcinoma in situ and Testicular Cancer Molecular and Endocrine Aspects**
Copenhagen, Denmark

*Further information from:*
Dr E Rajpert-De Meyts, Department of Growth and Reproduction, The National University Hospital, 9 Blogdamavej, 2100 Copenhagen, Denmark. Tel: +45 35 45 55 15 Fax: +45 35 45 60 54 E-mail: erm@rh.dk

*20–21 May 1997*

**7th International Symposium on Benign Breast Disease**
Royal Society of Medicine, London, UK

Symposium will cover the whole spectrum of new work in benign breast disease. Topics include: breast referrals in primary care; organization of the specialist breast clinic; new trials of mastalgia therapy; breast cancer prevention/high risk patient management; psychology and benign disease; the interface between gynaecology and benign disease; HRT and the breast; role of the breast care nurse/breast care physician; legal problems of breast diagnosis.

*Further information from:*
Georgina Pinnington, BBD7 Administration Office, Blythe Hall, 100 Blythe Road, London W14 0HB. Fax: 0171 371 4598

*7–11 September 1997*

**International Congress on Chronobiology**
Paris, France

*Further information from:*
Pr Yvan Touitou, Conference President, Internaitonal Congress on Chronobiology, Service de Biochimie Médicale, Faculté de Médecine Pitié-Salpêtrère, 91 boulevard de l' Hôpital, 75634 Paris Cedex 13, France. Tel: (+33-01) 40 77 96 63 Fax: (+33-01) 40 77 96 65 E-mail: touitou@ccr.jussleu.fr

*14–18 September 1997*

**The European Cancer Conference**
Hamburg, Germany

*Further information from:*
Luc Hendrickx, Conference and Finance Manager, ECCO 9 Secretariat, FBCS - Conference Unit, Avenue E Mounier 83, B-1200 Brussels. Tel: +32 (2) 775 02 02 Fax: +32 (2) 775 02 00

*24–26 September 1997*

**European Radiation Research '97, the Annual Meeting of the Association for Radiation Research and the 28th Annual Meeting of the European Society for Radiation Biology**
Oxford, UK

*Further information from:*
Dr Mohi Rezvani, Research Institute (University of Oxford), Churchill Hospital, Headington, Oxford OX3 7LJ, UK.
Tel: +44 (0)1865 225851 Fax: +44 (0)1865 225847
Email: mohi.rezvani.ox.ac.uk

*27–28 November 1997*

**Royal College of Physicians of Edinburgh – Consensus Conference on Platelet Transfusion**
Edinburgh, UK

*Further information from:*
Education, Audit and Research Department, Royal College of Physicians of Edinburgh, 9 Queen Street, Edinburgh EH2 1JQ, UK. Tel: +44 131 225 7324 Ext. 235. Fax: +44 131 220 4393.
Email: M.farquhar @ icpe.ac.uk

*20–24 June 1998*

**Second Cuneo Lung Cancer Conference**
Terme di Valdieri, Italy

*Further information from:*
Cuneo Lung Cancer Study Group (CuLCaSG), Via Romita, 15 12011 Borgo S Dalmazzo, Cuneo, Italy. Tel: 39171 260065